# Oral health care use characteristics in a limited sample of Medicare Advantage beneficiaries, Medicare Advantage Encounter data 2021

**DOI:** 10.1016/j.adaj.2025.03.005

**Published:** 2025-04-23

**Authors:** Darien J. Weatherspoon, Susan Hutfless, John F. Moeller, Richard J. Manski

**Affiliations:** assistant professor, Department of Dental Public Health, School of Dentistry, University of Maryland, Baltimore, MD.; lead research partner, Custom Data Shop, Conshohocken, PA.; research professor, Department of Dental Public Health, School of Dentistry, University of Maryland, Baltimore, MD.; professor and the chair, Department of Dental Public Health, School of Dentistry, University of Maryland, Baltimore, MD.

**Keywords:** Medicare Advantage, older adults, dental care use, inequities

## Abstract

**Background.:**

In contrast to the limited dental benefits available through traditional Medicare, most Medicare Advantage (MA) plans offer dental care services to eligible beneficiaries. MA Encounter data were made available to better understand the use of health services by MA beneficiaries. The authors’ objectives were to determine the degree to which dental care use data are available in the MA Encounter data source and understand the characteristics associated with dental care use in a sample of MA beneficiaries.

**Methods.:**

The 2021 Centers for Medicare & Medicaid Services Master Beneficiary Summary File, Plan Benefit Package, and Encounter data sources were analyzed. The primary outcome was receipt of at least 1 dental care service during the year, identified by a *CDT 2021: Current Dental Terminology* code. Bivariate analyses and a multivariable logistic regression model were used to assess the association between dental care use and sociodemographic factors in a sample of MA beneficiaries.

**Results.:**

A total of 23,725 MA beneficiaries in 21 plans met the inclusion criteria. Overall, 47% of beneficiaries in the sample had a *CDT 2021: Current Dental Terminology* code–recorded dental care use event. Beneficiaries used a range of comprehensive dental care services. Age, dual-eligibility status (ie, eligible for both Medicare and Medicaid), Medicare qualification reason, and plan type were associated with dental care use.

**Conclusions.:**

Dental care use data were available for only a small subset of plans within the MA Encounter data source. Select beneficiary characteristics were associated with dental care use in a limited MA beneficiary sample.

**Practical Implications.:**

There is a need for MA plans to enhance reporting of supplemental dental care use data to better understand whether MA is facilitating use of oral health care for the full beneficiary population.

Oral health is inextricably related to both overall health and well-being,^1^ and this relationship is especially evident among older adults, who have a higher prevalence of chronic conditions.^2–4^ Over time, older adults are retaining more of their natural teeth.^5^ Although this is a positive health trend, their teeth remain susceptible to oral diseases without appropriate oral health care maintenance. In addition, older adults possess unique risk factors for oral diseases, including medication-induced xerostomia, which makes having access to regular oral health care especially important.^6,7^

Despite the need for regular access to oral health care in this population, traditional Medicare fee-for-service (FFS), which many adults older than 65 years rely on for health care insurance, does not provide routine dental benefits to beneficiaries.^8^ Under limited circumstances when oral health care is determined to be inextricably linked to Medicare-covered medical procedures or specific medical conditions, Medicare FFS may cover select dental care services for eligible beneficiaries.^8^

In contrast to the limited dental benefits available through Medicare FFS, Medicare Advantage (MA) plans are offered by private insurance companies and most plans offer access to oral health care services as a supplemental benefit.^9^ Researchers have reported that nearly 94% of MA beneficiaries have access to dental care services through their MA plans.^9^ MA enrollment has increased considerably over time, with more than one-half of Medicare beneficiaries now enrolled in MA.^10^ Understanding the degree to which dental care services are being used is important for determining whether MA is serving the dental needs of its beneficiaries. Determining whether MA is facilitating equity in dental care service use for all members of the MA beneficiary population is also important because oral health inequities have been observed in older adults.^5,11,12^

MA Encounter data have been made available to researchers to better understand health care services use in the MA population. MA Encounter records contain information similar to that in claims, such as records of health care services provided.^13^ To our knowledge, MA Encounter data have not been used to assess dental use in the MA population. A primary challenge to using these data is the limited availability of data on supplemental benefits that are not included as part of traditional Medicare, such as dental care services. A report from the US Government Accountability Office found that the lack of complete data on supplemental benefits use is the result of incomplete reporting of these data by MA plans.^14^

To gain insights into dental care use in the MA population using the MA Encounter data source, we conducted an exploratory study with the following objectives: determine the degree to which dental care use data are available in the MA Encounter data source, describe characteristics of plans with adequate dental care use data, describe dental care use according to sociodemographic characteristics among a sample of beneficiaries in these plans, and understand what types of dental care services are being used by beneficiaries.

## METHODS

The Strengthening the Reporting of Observational Studies in Epidemiology guidelines were used for reporting the methodology and results of our study (eTable 1, available online at the end of this article).^15^

### Study design and data source

We analyzed dental care use of beneficiaries enrolled in MA plans using a cross-sectional study design. Data sources used to conduct the study analyses included 2021 Centers for Medicare & Medicaid Services (CMS) Master Beneficiary Summary File, Plan Benefit Package (PBP), and MA Encounter data.^16–18^ The Master Beneficiary Summary File contains data on beneficiary sociodemographic characteristics.^16^ The PBP contains quarterly data on benefits covered by MA plans.^17^ After observing minimal changes in coverage across quarters according to plan, we used the quarter 4 PBP data as representative for the entire calendar year. Encounter data contain MA records for services (eg, procedures and diagnoses) rendered by professional providers, such as physicians and dentists.^18^

### Study population

**Figure 1.**
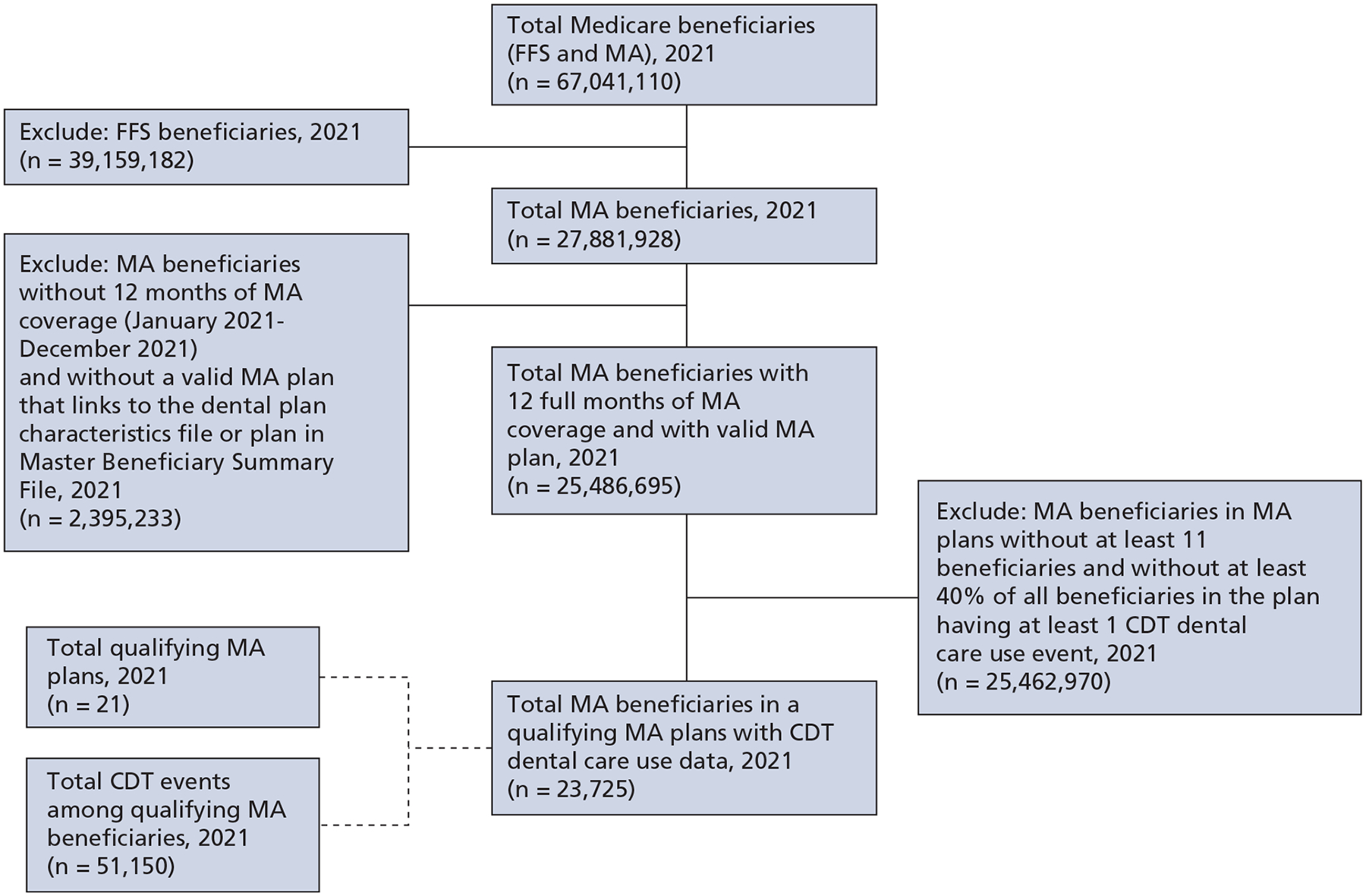
Flowchart of inclusion and exclusion criteria for analytic sample. CDT: *CDT 2021: Current Dental Terminology*.^19^ FFS: Fee-for-service. MA: Medical Advantage.

The initial inclusion criteria for our study’s analytic sample included 2021 MA beneficiaries with 12 months of Part C (MA) coverage (January 2021-December 2021) and with a valid MA plan that linked to the PBP (Figure 1), for which there were a total of 25,486,695 MA beneficiaries. To determine the availability of dental care use data within the MA Encounter Data source, the Carrier (Encounter) file was queried and a total of 537,771 *CDT 2021: Current Dental Terminology* (CDT)^19^ code events were identified for 141,781 MA beneficiaries. Because there are incomplete dental care use MA Encounter data,^13,14,20^ analyses were restricted to beneficiaries enrolled in MA plans in which at least 40% of all beneficiaries in the plan had at least 1 CDT dental care use event. This use threshold was based on previous survey data indicating that slightly more than 40% of MA beneficiaries reported using dental care services each year.^21,22^ To protect beneficiaries’ confidentiality, CMS data use agreements require the suppression of counts with a value of fewer than 11, so beneficiaries in MA plans with fewer than 11 total beneficiaries were excluded from analyses.^23^ There was a total of 21 MA plans identified that met these criteria, resulting in an analytic sample of 23,725 MA beneficiaries. Characteristics of these plans, including generosity of dental benefits they provided, were described using variables in the PBP.

### Primary outcome and covariates

The primary outcome of dental care service use was defined as the receipt of at least 1 dental care service during the year, identified by a CDT code within the Carrier (Encounter) data file. The American Dental Association maintains CDT codes, which are categorized as diagnostic (D0100-D0999); preventive (D1000-D1999); restorative (D2000-D2999); endodontics (D3000-D3999); periodontics (D4000-D4999); prosthodontics, removable (D5000-D5899); maxillofacial prosthetics (D5900-D5999); implant services (D6000-D6199); prosthodontics, fixed (D6200-D6999); oral and maxillofacial surgery (D7000-D7999); orthodontics (D8000-D8999); and adjunctive general services (D9000-D9999).^19^

Sociodemographic factors analyzed included age (< 65 years, 65–74 years, ≥ 75 years), sex (male, female), race and ethnicity (non-Hispanic American Indian or Alaska Native, non-Hispanic Asian, non-Hispanic Black, non-Hispanic White, Hispanic, other races and ethnicities, unknown race and ethnicity), dual-eligible status (ie, eligible for both Medicare and Medicaid), original reason for Medicare qualification (age, disability, end-stage kidney disease), and plan type. Due to small counts for many racial and ethnic groups in the sample, race and ethnicity were dichotomized into non-White or Hispanic and non-Hispanic White. Because 10 of the 21 plans meeting inclusion criteria were Program of All-Inclusive Care for the Elderly (PACE)^24^ plans, plan type was dichotomized into PACE and all other plan types.

### Data analyses

Characteristics of beneficiaries included in the analytic sample were described using univariate frequencies and percentages. χ^2^ statistics were used to compare the proportion of beneficiaries with and without CDT-recorded dental care use according to beneficiary sociodemographic factors. A multivariable logistic regression model was used to estimate odds ratios (ORs) for having a CDT-recorded dental care use encounter according to beneficiary sociodemographic factors, controlling for all other covariates. The prevalence of dental care services used by CDT category was examined and categorized as diagnostic (D0100-D0999), preventive (D1000-D1999), restorative (D2000-D2999), and specialty dental care services (D3000-D9999).^19^ Sensitivity analyses were conducted to determine whether characteristics associated with dental cae use and OR estimates remained similar when the inclusion criteria were modified to allow for the analysis of a larger sample. For these sensitivity analyses, dental care use was examined among beneficiaries in MA plans in which at least 30% of all beneficiaries in the plan had at least 1 CDT dental care use event, resulting in 126,199 beneficiaries. Sensitivity analyses were also conducted to see whether the OR estimates remained similar when beneficiaries younger than 65 years (1,441) were removed from the analytic sample, resulting in 22,284 beneficiaries for sensitivity analyses. The level of statistical significance used for our study was *P* < .05. SAS software, Version 9.4 (SAS Institute) was used to perform all study analyses.

### Protection of human participants

Our study was reviewed by the University of Maryland, Baltimore institutional review board (HP-00105777) and was determined to be exempt (category 4). In compliance with the data use agreement with the CMS, Custom Data Shop performed all analyses in the Virtual Data Research Center and provided investigators with aggregate study data to format and interpret.

## RESULTS

Of the 21 MA plans meeting inclusion criteria, 10 were PACE plans, 5 were preferred provider organization plans, 3 were health maintenance organization plans, 2 were health maintenance organization point of service plans, and 1 was a cost plan. The prevalence of dental care use among beneficiaries within these plans ranged from 41% through 75% (data not shown). No information on the generosity of dental benefits provided was available within the PBP for the 10 PACE plans. Among the remaining 11 plans, 10 offered preventive dental care services, with 9 providing mandatory coverage for oral examinations, prophylaxis, fluoride treatment, and dental radiographs. The remaining plan offering preventive dental care services provided mandatory coverage for all preventive services except fluoride treatment. These 10 plans also offered comprehensive dental care services, with 9 plans providing mandatory coverage for diagnostic, restorative, endodontics, periodontics, extractions, prosthodontics, other oral and maxillofacial surgery, and nonroutine services. The remaining plan offering comprehensive dental care services provided mandatory coverage for all comprehensive services except nonroutine services. One plan did not offer preventive or comprehensive dental care services.

Among 23,725 MA beneficiaries in the 21 MA plans meeting our study inclusion criteria, most beneficiaries (93.93%) were 65 years or older and there were more female (56.39%) than male beneficiaries (Table 1). The study sample overwhelmingly consisted of beneficiaries who were White (86.65%); American Indian or Alaska Native, Asian, Black, Hispanic, and other and unknown races and ethnicities comprised 13.35% of the sample. Similarly, the study sample overwhelmingly consisted of beneficiaries who did not have dual-eligible status (77.38%) and who qualified for Medicare on the basis of age (83.63%). A total of 80.30% of beneficiaries in our study sample were enrolled in non-PACE plans and 19.70% were enrolled in PACE plans. Compared with the full MA population, the analytical sample differed in that it had a smaller proportion of beneficiaries who were younger than 65 years, were part of a racial or ethnic minority group, and qualified for Medicare on the basis of disability or end-stage kidney disease. The analytic sample also had a substantially higher proportion of beneficiaries in PACE plans than the full MA population.

Overall, 46.93% of MA beneficiaries in the sample had a CDT-recorded dental care use event in 2021 (Table 2). Bivariate associations between dental care use and sociodemographic characteristics indicated that beneficiaries aged 65 through 74 years (49.21%) and 75 years and older (45.56%) had a higher prevalence of dental care use than beneficiaries younger than 65 years (37.54%). Female beneficiaries (48.24%) had a slightly higher prevalence of dental care use than male beneficiaries (45.22%). The prevalence of use between non-Hispanic White and non-White/Hispanic beneficiaries did not differ. Beneficiaries who were not dual-eligible (47.43%) had only a slightly higher prevalence of use than those who were dual-eligible (45.20%). Those beneficiaries who qualified for Medicare on the basis of age (48.28%) had a higher prevalence of use than those who qualified for Medicare on the basis of disability or end-stage kidney disease (39.99%). Finally, beneficiaries in PACE plans (48.33%) had a similar prevalence of use as those in other plan types (46.58%). Distributions of dental care use across sociodemographic categories were also displayed using column percentages and are presented in eTable 2 (available online at the end of this article).

Using fully adjusted multivariable logistic regression models to assess independent association between sociodemographic characteristics and dental care use in the analytic sample, we found that those beneficiaries younger than 65 years and 75 years and older had lower odds of use than beneficiaries aged 65 through 74 years (Table 3). Female beneficiaries had slightly higher odds of use than male beneficiaries (OR, 1.13; 95% CI, 1.08 to 1.19). Non-White or Hispanic and non-Hispanic White beneficiaries did not differ significantly in their odds for dental care use. Dual-eligible beneficiaries (OR, 0.40; 95% CI, 0.34 to 0.48) had significantly lower odds of use than non–dual-eligible beneficiaries, and those beneficiaries who qualified for Medicare on the basis of disability or end-stage kidney disease (OR, 0.73; 95% CI, 0.67 to 0.80) had significantly lower odds of use than those beneficiaries who qualified for Medicare on the basis of age. Finally, beneficiaries in PACE plans had significantly higher odds of use than beneficiaries enrolled in non-PACE plans (OR, 2.86; 95% CI, 2.38 to 3.44).

**Figure 2.**
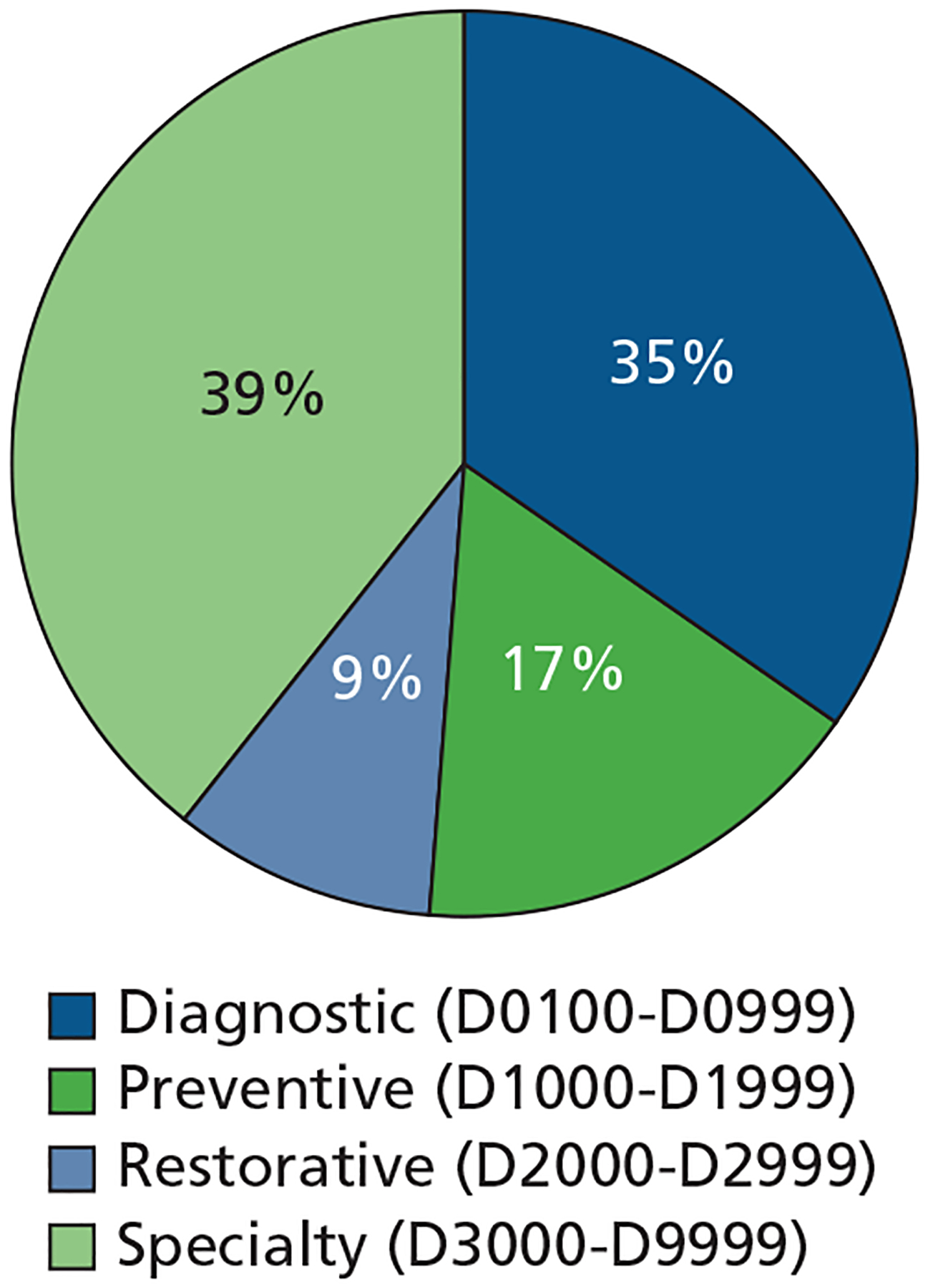
Dental care use events by *CDT 2021: Current Dental Terminology*^19^ code category among those in eligible Medicare Advantage plans (51,150), Medicare Advantage Encounter data 2021.^18^

Among 51,150 CDT codes in the analytic sample (Figure 2), 39% were for specialty dental care services (D3000-D9999), 35% were for diagnostic dental care services (D0100-D0999), 17% were for preventive dental care services (D1000-D1999), and 9% were for restorative dental care services (D2000-D2999).^19^ Among the total 537,771 CDT codes identified in the MA Encounter data, 41% were for diagnostic dental care services (D0100-D0999), 33% were for specialty dental care services (D3000-D9999), 17% were for preventive dental care services (D1000-D1999), and 9% were for restorative dental care services (D2000-D2999).

Sensitivity analyses with a larger sample size found overall similar patterns of dental care use according to beneficiary sociodemographic characteristics, with slight differences noted in ORs for use across factors (eTables 3 and 4, available online at the end of this article). Significantly higher odds for use remained for beneficiaries enrolled in PACE plans, controlling for other factors (OR, 3.68; 95% CI, 3.37 to 4.01). Finally, when logistic regression analyses were restricted to beneficiaries 65 years and older (22,284) for sensitivity analyses, the OR effect estimates did not change appreciably (data not shown).

## DISCUSSION

Our study findings provide data to further describe the degree to which dental care use MA Encounter data are available and characteristics of plans with higher levels of dental care use MA Encounter data. Our results also provide insights on characteristics associated with use and types of dental care services being used in a limited MA sample.

MA plans have been explored as a way to increase use of oral health care for the Medicare population because, unlike traditional Medicare, most MA plans offer beneficiaries eligibility for dental care services as a supplemental benefit.^9^ Results of our analysis of available CDT codes,^19^ both in our sample and within the entire MA Encounter data source, showed that a full range of comprehensive dental care services were used. Of the total CDT codes identified in the 2021 MA Encounter data, 42% were for restorative and specialty dental care services, in addition to diagnostic (41%) and preventive (17%) dental care services. Although encounter dental records are not complete,^13,14,20^ our findings indicate that beneficiaries are using a full range of comprehensive dental care services through MA.

Focusing on dental care service use, we confirmed previous findings on the limited availability of MA Encounter data for supplemental MA services.^13,14,20^ More specifically, we identified only 21 MA plans in the MA Encounter data source, with data showing that at least 40% of beneficiaries within the plan had a dental care use event. Roughly one-half of these plans were MA PACE plans, and the remaining were MA plans with generous dental benefits (ie, providing mandatory comprehensive and preventive dental coverage). These characteristics could have influenced their reporting of MA Encounter dental care use data. We also likely analyzed dental care use in a group of plans and beneficiaries that is not representative of the full MA population. For example, we found that beneficiaries in MA PACE plans were substantially overrepresented in our sample (20%) compared with their proportion of the full MA population (0.2%).

PACE plans are only available in select regions, and, in addition to covering health services, they provide additional care coordination services for qualifying beneficiaries who can live safely in the community but need the types of care typically provided in nursing homes.^24^ The characteristics of beneficiaries with MA PACE plans and the additional services that these plans provide could explain the strong independent association observed between having an MA PACE plan and dental care use in both our primary and sensitivity analyses, when controlling for other sociodemographic factors. Controlling for other factors, we also observed greater odds for use among those aged 65 through 74 years, female, non–dual-eligible, and qualifying for Medicare due to age (rather than disability) among our sample. However, care must be taken when interpreting these findings, due to our limited sample size and the characteristics of plans meeting our inclusion criteria. Therefore, our analyses should be replicated in a larger, more generalizable MA sample as more MA Encounter data become available, to better elucidate the independent effect of these sociodemographic factors on dental care use in the full MA population.

To gain a comprehensive understanding of dental care use in the MA population through the MA Encounter data source, complete data will be necessary. On the basis of recommendations from the US Government Accountability Office, in February of 2024, CMS took 2 steps to help ensure future complete MA Encounter data for supplemental health care services, such as dental care services.^14^ CMS released guidance to clarify that MA plans are required to submit MA Encounter data for all supplemental benefits provided to their MA enrollees, in addition to Medicare Part A– and Part B–covered items and services.^14^ CMS also detailed changes that have been made to address MA plans’ challenges in submitting MA Encounter data for supplemental benefits as well as guidance to MA plans on how to submit MA Encounter data.^14^ These updated CMS instructions to MA plans apply to supplemental benefits provided in 2024. Therefore, it is expected that future MA Encounter data will have complete data needed to comprehensively assess dental care use and access to dental care services for the entire MA population.

Understanding whether MA is improving access to and use of oral health care services for the entire MA population is important because previous analyses by Simon and Cai^21^ using Medical Expenditure Panel Survey data found that, despite the availability of dental benefits through MA, MA dental care use does not differ much from Medicare FFS dental care use, which could be due to high out-of-pocket costs associated with plans or lack of awareness of benefits. Understanding whether MA is facilitating access to oral health care services for populations that have been historically underserved is also important. Over time, enrollment in MA has been greatest among people in racial and ethnic minority groups and those who have dual-eligible status (a proxy for having lower income); researchers have found that beneficiaries who are in racial and ethnic minority groups, have dual-eligible status, and qualified for Medicare due to disability status are more likely to have MA plans offering dental benefits.^9,25–27^ Therefore, MA could improve disparities in access to oral health care previously reported in the Medicare population.^28^ Although we found some differences in use according to sociodemographic characteristics in our sample, given our restricted sample and small sample sizes for many demographic groups, we were not able to make inferences about the degree to which inequities in dental care use may exist across the full MA population. Having access to complete MA Encounter data will allow researchers to comprehensively assess dental care access and use through MA to determine whether the program is meeting the dental needs of all its beneficiaries.

There are limitations to our study. As mentioned above, our study findings are generalizable only to the limited sample of MA beneficiaries analyzed and not to the entire MA population. The sample we analyzed was underrepresented by racial and ethnic minority groups compared with the full MA population and the small samples sizes for beneficiaries in many racial and ethnic groups required collapsing these beneficiaries into 1 category, thereby resulting in less granular data on use by race and ethnicity.^29^ In addition, compared with the full MA population, our sample was substantially overrepresented by beneficiaries in MA PACE plans, a plan type and patient population that could have affected the use patterns we observed. Furthermore, analyzing dental care use among dual-eligible beneficiaries using only MA Encounter data likely results in underreporting their use because Medicaid may cover their dental care services.^30^ Finally, even by means of restricting our analyses to plans with higher levels of dental care use, we are unable to confirm whether complete dental care use data were available for beneficiaries in these plans, given limitations with MA Encounter data reporting.^13,14,20^ Missing data could have affected study results. Therefore, future studies are warranted to replicate analyses when complete dental MA Encounter data for the full MA population become available.

The strength of our study is that we are, to our knowledge, the first to examine and describe dental care use for the MA population using MA Encounter data, a rich data source that can address some limitations of use analyses that rely on self-reporting by beneficiaries.^13,22^ Our findings also provide policy makers and researchers with a more detailed understanding of the degree to which dental care service use data are available in the MA Encounter data source. In addition, we provided preliminary data on characteristics associated with dental care use for a sample of the MA population, which can be used to inform future analyses when full MA Encounter data become available.

## CONCLUSIONS

We found that MA beneficiaries are using a full range of comprehensive dental care services. We also found that dental care use data within the CMS MA Encounter data source are limited, which prevents researchers from analyzing use across the entire MA population. Among a limited sample of MA beneficiaries, we identified factors associated with dental care use. However, complete dental care MA Encounter data for the full MA beneficiary population is needed to comprehensively analyze dental care use to determine whether MA is helping improve access to oral health care and facilitate equity for its beneficiaries. In the future, researchers should also investigate the multilevel factors, including behavioral and community-level factors, that can influence dental care use and access to care in the Medicare population.^31,32^

## Supplementary Material

1

Supplemental data related to this article can be found at: https://dx.doi.org/10.1016/j.adaj.2025.03.005.

## Figures and Tables

**Table 1.** Frequencies and column percentages* of beneficiaries according to select demographic characteristics among those in eligible MA^†^ plans^‡^ and for the full MA population, 2021.^§^

CHARACTERISTIC	SAMPLE TOTAL, NO. (%) (n = 23,725)	MA TOTAL WITH 12 MONTHS OF MA COVERAGE AND PLAN JOINED TO DENTAL CHARACTERISTICS PLANS, NO. (%) (n = 25,486,695)
**Age, Y**		
<65	1,441 (6.07)	3,198,915 (12.55)
65–74	12,047 (50.78)	12,192,774 (47.84)
≥75	10,237 (43.15)	10,095,006 (39.61)
**Sex**		
Female	13,378 (56.39)	14,407,800 (56.53)
Male	10,347 (43.61)	11,078,894 (43.47)
**Race and Ethnicity**		
Non-Hispanic White	20,558 (86.65)	19,103,779 (74.96)
Non-White or Hispanic^¶^	3167 (13.35)	6,382,916 (25.04)
**Dual-Eligible** ^#^		
No	18,358 (77.38)	19,852,573 (77.89)
Yes	5,367 (22.62)	5,634,122 (22.11)
**Original Reason for Medicare Qualification**		
Age	19,842 (83.63)	19,244,858 (75.51)
Disability or end-stage kidney disease**	3,883 (16.37)	6,241,837 (24.49)
**Plan Type**		
Program of All-Inclusive Care for the Elderly	4,674 (19.70)	47,382 (0.19)
All other plan types^††^	19,051 (80.30)	25,439,313 (99.81)

* Percentages may not add to 100% due to rounding.

† MA: Medicare Advantage.

‡ Subset of beneficiaries in 21 MA plans meeting study inclusion criteria.

§ Source: Research Data Assistance Center, Centers for Medicare & Medicaid Services.^16^

¶ Beneficiaries who are American Indian or Alaska Native, Asian, Black, Hispanic, other races and ethnicities, and unknown races and ethnicities were combined into 1 category due to small cell counts.

# Eligible for both Medicare and Medicaid.

** Beneficiaries qualifying for Medicare due to disability alone, end-stage kidney disease alone, and disability and end-stage kidney disease alone were combined into 1 category due to small cell counts, which would have required cell suppression.

†† Beneficiaries in Preferred Provider Organization, Health Maintenance Organization, Health Maintenance Organization Point of Service, and Cost plans were combined into 1 category.

**Table 2.** Frequencies and row percentages of beneficiaries according to select demographic characteristics and dental care use among those in eligible Medicare Advantage plans,* 2021.^†^

CHARACTERISTIC	NO CDT^‡^-RECORDED DENTAL CARE USE EVENT, NO. (%) (n = 12,592 [53.07%])	CDT-RECORDED DENTAL CARE USE EVENT,^§^ NO. (%) (n = 11,133 [46.93%])	TOTAL, NO. (n = 23,725)	*P* VALUE^¶^
**Age, Y**				
<65	900 (62.46)	541 (37.54)	1,441	
65–74	6,119 (50.79)	5,928 (49.21)	12,047	<.0001
≥75	5,573 (54.44)	4,664 (45.56)	10,237	
**Sex**				
Female	6,924 (51.76)	6,454 (48.24)	13,378	
Male	5,668 (54.78)	4,679 (45.22)	10,347	<.0001
**Race and Ethnicity**				
Non-White or Hispanic^#^	1,687 (53.27)	1,480 (46.73)	3,167	
Non-Hispanic White	10,905 (53.05)	9,653 (46.95)	20,558	.8149
**Dual-Eligible** **				
No	9,651 (52.57)	8,707 (47.43)	18,358	
Yes	2,941 (54.80)	2,426 (45.20)	5,367	.0041
**Original Reason for Medicare Qualification**				
Age	10,262 (51.72)	9,580 (48.28)	19,842	
Disability or end-stage kidney disease^††^	2,330 (60.01)	1,553 (39.99)	3,883	<.0001
**Plan Type**				
Program of All-Inclusive Care for the Elderly	2,415 (51.67)	2,259 (48.33)	4,674	
All other plan types^‡‡^	10,177 (53.42)	8,874 (46.58)	19,051	.0314

* Subset of beneficiaries in 21 Medicare Advantage plans meeting study inclusion criteria.

† Source: Research Data Assistance Center, Centers for Medicare & Medicaid Services.^16,18^

‡ CDT: *CDT 2021: Current Dental Terminology*.^19^

§ CDT-recorded dental care use event is defined as a beneficiary having a recorded CDT code identified through Medicare Advantage Encounter claims.

¶ *P* value χ^2^ test of association between CDT-recorded dental care use and demographic characteristic categories.

# Beneficiaries who are American Indian or Alaska Native, Asian, Black, Hispanic, other races and ethnicities, and unknown races and ethnicities were combined into 1 category due to small individual cell counts.

** Eligible for both Medicare and Medicaid.

†† Beneficiaries qualifying for Medicare due to disability alone, end-stage kidney disease, and disability and end-stage kidney disease were combined into 1 category due to small individual cell counts, which would have required cell suppression.

‡‡ Beneficiaries in Preferred Provider Organization, Health Maintenance Organization, Health Maintenance Organization Point of Service, and Cost plans were combined into one category.

**Table 3.** Odds ratios (95% CIs) of *CDT 2021: Current Dental Terminology*^19^ code–recorded dental care use according to select demographic characteristics among beneficiaries in eligible Medicare Advantage Plans,* 2021.^†^

CHARACTERISTIC	UNADJUSTED ODDS RATIO (95% CI)	ADJUSTED ODDS RATIO^‡^ (95% CI)
**Age, Y [Reference, 65–74]**		
<65	0.62 (0.55 to 0.69)	0.87 (0.76 to 0.99)
≥75	0.86 (0.82 to 0.91)	0.83 (0.79 to 0.87)
**Sex [Reference, Male]**		
Female	1.13 (1.07 to 1.19)	1.13 (1.08 to 1.19)
**Race and Ethnicity [Reference, Non-Hispanic White]**		
Non-White or Hispanic^§^	0.99 (0.92 to 1.07)	0.93 (0.85 to 1.01)
**Dual-Eligible**^¶^ **[Reference, No]**		
Yes	0.91 (0.86 to 0.97)	0.40 (0.34 to 0.48)
**Original Reason for Medicare Qualification [Reference, Age]**		
Disability or end-stage kidney disease^#^	0.71 (0.67 to 0.77)	0.73 (0.67 to 0.80)
**Plan Type [Reference, All Other Plan Types** ** **]**		
Program of All-Inclusive Care for the Elderly	1.07 (1.01 to 1.14)	2.86 (2.38 to 3.44)

* Subset of beneficiaries in 21 Medicare Advantage plans meeting study inclusion criteria.

† Source: Research Data Assistance Center, Centers for Medicare & Medicaid Services.^16,18^

‡ Fully adjusted logistic regression model, adjusting for all covariates.

§ Beneficiaries who are American Indian or Alaska Native, Asian, Black, Hispanic, other races and ethnicities, and unknown races and ethnicities were combined into 1 category due to small individual cell counts.

¶ Eligible for both Medicare and Medicaid.

# Beneficiaries qualifying for Medicare due to disability alone, end-stage kidney disease alone, and disability and end-stage kidney disease were combined into 1 category due to small individual cell counts, which would have required cell suppression.

** Beneficiaries in Preferred Provider Organization, Health Maintenance Organization, Health Maintenance Organization Point of Service, and Cost plans were combined into 1 category.

## ABBREVIATION KEY

CDT: *CDT 2021: Current Dental Terminology*
CMS: Centers for Medicare & Medicaid Services
FFS: Fee-for-service
MA: Medicare Advantage
PACE: Program of All-Inclusive Care for the Elderly
PBP: Plan Benefit Package
