# Supplementary material for: Oral health care use characteristics in a limited sample of Medicare Advantage beneficiaries, Medicare Advantage Encounter data 2021
Source: J Am Dent Assoc. Author manuscript; Available in PMC 2025 Jun 12. (PMC12160556; doi:10.1016/j.adaj.2025.03.005)
Supplement: 1 [file NIHMS2087179-supplement-1.pdf]

eTable 1. Strengthening the Reporting of Observational Studies in Epidemiology statement[15]: checklist of items that should be included in reports of cross-sectional studies.*

ARTICLE ELEMENT	ITEM NO.	RECOMMENDATION	PAGE NO.
**Title and Abstract**	1	(a) Indicate the study's design with a commonly used term in the title or the abstract<br>(b) Provide in the abstract an informative and balanced summary of what was done and what was found	458<br>458
**Introduction**			
Background and rationale	2	Explain the scientific background and rationale for the investigation being reported	458, 459
Objectives	3	State specific objectives, including any prespecified hypotheses	459
**Methods**			
Study design	4	Present key elements of study design early in the article	459
Setting	5	Describe the setting, locations, and relevant dates, including periods of recruitment, exposure, follow-up, and data collection	459, 460
Participants	6	(a) Give the eligibility criteria, and the sources and methods of selection of participants	459, 460
Variables	7	Clearly define all outcomes, exposures, predictors, potential confounders, and effect modifiers. Give diagnostic criteria, if applicable	460
Data sources and measurement	8†	For each variable of interest, give sources of data and details of methods of assessment (measurement). Describe comparability of assessment methods if there is more than 1 group	459, 460
Bias	9	Describe any efforts to address potential sources of bias	459
Study size	10	Explain how the study size was arrived at	459, 460
Quantitative variables	11	Explain how quantitative variables were handled in the analyses. If applicable, describe which groupings were chosen and why	460, 461
Statistical methods	12	(a) Describe all statistical methods, including those used to control for confounding<br>(b) Describe any methods used to examine subgroups and interactions<br>(c) Explain how missing data were addressed<br>(d) If applicable, describe analytical methods taking account of sampling strategy<br>(e) Describe any sensitivity analyses	460, 461<br>NA‡<br>NA<br>NA<br>461
**Results**			
Participants	13†	(a) Report numbers of individuals at each stage of study, eg, numbers potentially eligible, examined for eligibility, confirmed eligible, included in the study, completing follow-up, and analyzed<br>(b) Give reasons for nonparticipation at each stage<br>(c) Consider use of a flow diagram	459, 460<br>459, 460<br>460
Descriptive data	14†	(a) Give characteristics of study participants (eg, demographic, clinical, social) and information on exposures and potential confounding variables<br>(b) Indicate number of participants with missing data for each variable of interest	462, 463<br>NA
Outcome data	15†	Report numbers of outcome events or summary measures	463, 464
Main results	16	(a) Give unadjusted estimates and, if applicable, confounder-adjusted estimates and their precision (eg, 95% CI). Make clear which confounders were adjusted for and why they were included<br>(b) Report category boundaries when continuous variables were categorized<br>(c) If relevant, consider translating estimates of relative risk into absolute risk for a meaningful time	463, 464<br>NA<br>NA
Other analyses	17	Report other analyses done, eg, analyses of subgroups and interactions, and sensitivity analyses	NA
**Discussion**			
Key results	18	Summarize key results with reference to study objectives	464

* An explanation and elaboration article discusses each checklist item and gives methodological background and published examples of transparent reporting. The Strengthening the Reporting of Observational Studies in Epidemiology checklist is best used in conjunction with this article (freely available on the web sites of *PLoS Medicine* at http://www.plosmedicine.org, *Annals of Internal Medicine* at http://www.annals.org, and *Epidemiology* at http://www.epidem.com). Information on the Strengthening the Reporting of Observational Studies in Epidemiology initiative is available at www.strobe-statement.org. † Give information separately for exposed and unexposed groups. ‡ NA: Not applicable.

eTable 1. Continued

ARTICLE ELEMENT	ITEM NO.	RECOMMENDATION	PAGE NO.
Limitations	19	Discuss limitations of the study, taking into account sources of potential bias or imprecision. Discuss both direction and magnitude of any potential bias	465, 466
Interpretation	20	Give a cautious overall interpretation of results considering objectives, limitations, multiplicity of analyses, results from similar studies, and other relevant evidence	464-466
Generalizability	21	Discuss the generalizability (external validity) of the study results	464-466
**Other Information**			
Funding	22	Give the source of funding and the role of the funders for the present study and, if applicable, for the original study on which the present article is based	466

eTable 2. Frequencies and column percentages of beneficiaries according to select demographic characteristics and dental care use among those in eligible Medicare Advantage plans,* 2021.†

CHARACTERISTIC	NO CDT‡-RECORDED DENTAL CARE USE EVENT, NO. (%) (n = 12,592)	CDT-RECORDED DENTAL CARE USE EVENT,§ NO. (%) (n = 11,133)	TOTAL, NO. (n = 23,725)	*P* VALUE¶
**Age, Y**				
< 65	900 (7.15)	541 (4.86)	1,441 (6.07)	< .0001
65-74	6,119 (48.59)	5,928 (53.25)	12,047 (50.78)	
≥ 75	5,573 (44.26)	4,664 (41.89)	10,237 (43.15)	
**Sex**				
Female	6,924 (54.99)	6,454 (57.97)	13,378 (56.39)	< .0001
Male	5,668 (45.01)	4,679 (42.03)	10,347 (43.61)	
**Race and Ethnicity**				
Non-White or Hispanic#	1,687 (13.40)	1,480 (13.29)	3,167 (13.35)	.8149
Non-Hispanic White	10,905 (86.60)	9,653 (86.71)	20,558 (86.65)	
**Dual-Eligible****				
No	9,651 (76.64)	8,707 (78.21)	18,358 (77.38)	.0041
Yes	2,941 (23.36)	2,426 (21.79)	5,367 (22.62)	
**Original Reason for Medicare Qualification**				
Age	10,262 (81.50)	9,580 (86.05)	19,842 (83.63)	< .0001
Disability or end stage kidney disease††	2,330 (18.50)	1,553 (13.95)	3,883 (16.37)	
**Plan Type**				
Program of All-Inclusive Care for the Elderly	2,415 (19.18)	2,259 (20.29)	4,674 (19.70)	.0314
All other plan types‡‡	10,177 (80.82)	8,874 (79.71)	19,051 (80.30)	

* Subset of beneficiaries in 21 Medicare Advantage plans meeting study inclusion criteria. † Source: Research Data Assistance Center, Centers for Medicare & Medicaid Services.[16,18] ‡ CDT: *CDT 2021: Current Dental Terminology*.[19] § CDT-recorded dental care use event is defined as a beneficiary having a recorded CDT code identified through Medicare Advantage Encounter claims. ¶ *P* value for χ^2 test of association between CDT-recorded dental care use and demographic characteristic categories. # Beneficiaries who are American Indian or Alaska Native, Asian, Black, Hispanic, other races and ethnicities, and unknown races and ethnicities were combined into 1 category due to small individual cell counts. ** Eligible for both Medicare and Medicaid. †† Beneficiaries qualifying for Medicare due to disability alone, end-stage kidney disease alone, and disability and end-stage kidney disease were combined into 1 category due to small individual cell counts, which would have required cell suppression. ‡‡ Beneficiaries in preferred provider organization, health maintenance organization, health maintenance organization point of service, and cost plans were combined into 1 category.

eTable 3. Frequencies and row percentages of beneficiaries according to select demographic characteristics and dental care use among those in eligible Medicare Advantage plans,* 2021.†

CHARACTERISTIC	NO CDT‡-RECORDED DENTAL CARE USE EVENT, NO. (%) (n = 78,507 [62.21%])	CDT-RECORDED DENTAL CARE USE EVENT,§ NO. (%) (n = 47,692 [37.79%])	TOTAL, NO. (n = 126,199)	*P* VALUE¶
**Age, Y**				
< 65	4,667 (71.76)	1,837 (28.24)	6,504	< .0001
65-74	35,807 (59.46)	24,413 (40.54)	60,220	
≥ 75	38,033 (63.95)	21,442 (36.05)	59,475	
**Sex**				
Female	43,040 (60.69)	27,877 (39.31)	70,917	< .0001
Male	35,467 (64.16)	19,815 (35.84)	55,282	
**Race and Ethnicity**				
Non-White or Hispanic#	6,088 (63.02)	3,573 (36.98)	9,661	.0885
Non-Hispanic White	72,419 (62.14)	44,119 (37.86)	116,538	
**Dual-Eligible****				
No	69,989 (61.69)	43,458 (38.31)	113,447	< .0001
Yes	8,518 (66.80)	4,234 (33.20)	12,752	
**Original Reason for Medicare Qualification**				
Age	66,103 (60.74)	42,727 (39.26)	108,830	< .0001
Disability or end-stage kidney disease††	12,404 (71.41)	4,965 (28.59)	17,369	
**Plan Type**				
Program of All-Inclusive Care for the Elderly	4,329 (56.49)	3,335 (43.51)	7,664	< .0001
All other plan types‡‡	74,178 (62.58)	44,357 (37.42)	118,535	

* Subset of beneficiaries in 65 Medicare Advantage plans in which at least 30% of beneficiaries had a *CDT 2021: Current Dental Terminology*[19] dental care use event and at least 11 beneficiaries in the plan. Used for a sensitivity analysis. † Source: Research Data Assistance Center, Centers for Medicare & Medicaid Services.[16,18] ‡ *CDT 2021: Current Dental Terminology*.[19] § CDT-recorded dental care use event is defined as a beneficiary having a recorded CDT code identified through Medicare Advantage Encounter claims. ¶ *P* value for χ^2 test of association between CDT-recorded dental care use and demographic characteristic categories. # Beneficiaries who are American Indian or Alaska Native, Asian, Black, Hispanic, other races and ethnicities, and unknown races and ethnicities were combined into 1 category due to small individual cell counts. ** Eligible for both Medicare and Medicaid. †† Beneficiaries qualifying for Medicare due to disability alone, end-stage kidney disease alone, and disability and end-stage kidney disease were combined into 1 category due to small individual cell counts, which would have required cell suppression. ‡‡ Beneficiaries in preferred provider organization, health maintenance organization, health maintenance organization point of service, and cost plans were combined into 1 category.

eTable 4. Odds ratios (and 95% CI) of *CDT 2021: Current Dental Terminology*[19]–recorded dental care use according to select demographic characteristics among beneficiaries in eligible Medicare Advantage Plans,* 2021.†

CHARACTERISTIC	UNADJUSTED ODDS RATIO (95% CI)	ADJUSTED ODDS RATIO‡ (95% CI)
**Age, Y [Reference, 65-74]**		
< 65	0.58 (0.55 to 0.61)	1.00 (0.94 to 1.08)
≥ 75	0.83 (0.81 to 0.85)	0.79 (0.77 to 0.81)
**Sex [Reference, Male]**		
Female	1.16 (1.13 to 1.19)	1.16 (1.13 to 1.19)
**Race and Ethnicity [Reference, Non-Hispanic White]**		
Non-White or Hispanic§	0.96 (0.92 to 1.01)	0.89 (0.85 to 0.93)
**Dual-Eligible¶ [Reference, No]**		
Yes	0.80 (0.77 to 0.83)	0.40 (0.37 to 0.43)
**Original Reason for Medicare Qualification [Reference, Age]**		
Disability or end-stage kidney disease#	0.62 (0.60 to 0.64)	0.60 (0.57 to 0.63)
**Plan Type [Reference, All Other Plan Types**]**		
Program of All-Inclusive Care for the Elderly	1.29 (1.23 to 1.35)	3.68 (3.37 to 4.01)

* Subset of beneficiaries in 65 Medicare Advantage plans in which at least 30% of beneficiaries had a *CDT 2021: Current Dental Terminology*[19] dental care use event and at least 11 beneficiaries were in the plan. Used for a sensitivity analysis. † Source: Research Data Assistance Center, Centers for Medicare & Medicaid Services.[16,18] ‡ Fully adjusted logistic regression model, adjusting for all covariates. § Beneficiaries who are American Indian or Alaska Native, Asian, Black, Hispanic, other races and ethnicities, and unknown races and ethnicities were combined into 1 category due to small individual cell counts. ¶ Eligible for both Medicare and Medicaid. # Beneficiaries qualifying for Medicare due to disability alone, end-stage kidney disease alone, and disability and end-stage kidney disease were combined into 1 category due to small individual cell counts, which would have required cell suppression. ** Beneficiaries in preferred provider organization, health maintenance organization, health maintenance organization point of service, and cost plans were combined into 1 category.
